# Chondral regeneration in femoroacetabular lesions is favoured using peripheral blood stem cells with hyaluronan‐based scaffold and micro‐drilling: A prospective cohort study

**DOI:** 10.1002/jeo2.70009

**Published:** 2024-08-30

**Authors:** Juan Eduardo Monckeberg, Claudio Rafols, Philipp Gerhard, Leticia Del Canto, Julio Rosales, Marco Antonio Verdugo, Cristobal Saez, Carlos De la Fuente

**Affiliations:** ^1^ Traumatologia, Clínica MEDS Santiago Chile; ^2^ Radiologia, Clínica MEDS Santiago Chile; ^3^ Exercise and Rehabilitation Sciences Institute, Postgraduate, Faculty of Rehabilitation Sciences Universidad Andres Bello Santiago de Chile Chile

**Keywords:** arthroscopy, chondral repair, hyalofast, stem cells, surgery preservation

## Abstract

**Purpose:**

To determine whether intra‐articular injections of peripheral blood stem cells (PBSC) with hyaluronan (HA)‐based scaffold improve articular cartilage regeneration in chondral injuries caused by mixed‐femoroacetabular impingement syndrome (FAIS) over a period longer than 24 months post‐hip arthroscopy.

**Methods:**

In this prospective cohort study, patients with mixed‐FAIS and chondral injury ≥ IIIB according to the International Cartilage Regeneration and Joint Preservation Society grade or III/IV of Konan/Haddad classification underwent intra‐articular injection of PBSC with an HA‐based scaffold and micro‐drillings during hip arthroscopy. The degree of chondral repair was measured at baseline and 5 years using the International Cartilage Repair Society morphologic score system (MSS) as the primary outcome. Pain was measured at baseline and 5 years using the Visual Analogue Scale for Pain (VAS Pain), and hip functionality was measured at baseline (presurgery), 6 months, 1 year, and 5 years using the Hip Outcome Score (HOS). The largest diameter of injury, median follow‐up, side effects, complications, and improvements were described. *T*‐test, ANOVA with multiple comparisons, and statistical power were estimated.

**Results:**

From initially 34 cases, 25 patients were enrolled. The median follow‐up was 5.1 ± 0.3 years. One patient (4%) reported a few side effects with filgrastim administration. No infection, tumours, or synovitis was reported. The largest diameters in zones two, three, and four were 12.4 ± 3.1 mm (*n* = 8), 13.5 ± 2.8 mm (*n* = 14), and 11.4 ± 1.9 mm (*n* = 3), respectively. Ninety‐two percent (23/25) of patients improved their outcomes. The MSS and HOS increased from 3.8 ± 1.1 to 9.6 ± 1.5 pts (*p* < 0.001) and from 65.5 ± 13.0 to 93.9 ± 2.4 pts (*p* < 0.001), respectively. The VAS‐Pain decreased from 5.3 ± 0.7 to 1.3 ± 0.6 mm (*p* < 0.001). The obtained a *posteriori* power‐size was 0.99.

**Conclusion:**

The intervention suggests a favourable impact on articular cartilage regeneration and clinical outcomes for hip chondral lesions in mixed‐FAIS injuries after a median follow‐up of 5.1 ± 0.3 years.

**Level of Evidence:**

Level IV.

**Abbreviations**
ANOVA: analysis of variance
FAIS: femoroacetabular impingement syndrome
HA: hyaluronan
HOS: hip outcome score
ICRS: the International Cartilage Repair Society
MRI: magnetic resonance imaging
MSS: morphologic score system
PBSC: peripheral blood stem cells
PRP: platelet‐rich plasma
VAS Pain: visual analogue scale for pain

## BACKGROUND

Repetitive trauma from femoroacetabular impingement syndrome (FAIS) [47] can cause full‐thickness defects and subchondral bone loss [14, 15, 34], with grade III/IV of the International Cartilage Repair Society (ICRS) classification representing the most severe chondral injury [30].

Although the hip cartilage has limited healing and regeneration capacity [20], cell therapy in preservation surgery helps in joint restoration [12, 17, 25]. In vitro, ex vivo, and in vivo studies support cell therapy [11, 12, 21, 26, 37], which has employed bone marrow stem components, collagen scaffolds, platelet‐rich fibrin glue scaffold, collagen gel and periosteum, or hydroxyapatite ceramic [11, 12, 21, 26, 37]. Recently, intra‐articular injections of peripheral blood stem cells (PBSC) have shown promising results in knee osteochondral injuries [32, 42]. Biodegradable hyaluronic acid (HA)‐based scaffolds following micro‐drilling also support hyaline‐like cartilage regeneration in grade IV cartilage knee ulcers [45], suggesting potential for femoroacetabular chondral injuries. Micro‐drilling, a less detrimental technique for subchondral bone than microfractures, enhances marrow stroma stimulation and biomechanical properties of tissue [24, 36].

The PBSC with HA‐based scaffold technique captures mesenchymal stem cells and supports their attachment, proliferation, and differentiation, filling the chondral defect [45]. The release of HA creates an embryonic‐like microenvironment to promote cartilage growth [45]. Unfortunately, progressive deterioration of the neoformed chondral tissue can occur at the lesion site 24–36 months after treatment [31]. Although clinical and radiological outcomes using PBSC with an HA‐based scaffold show positive results in knee injuries and may be comparable to bone marrow cell use [11, 38, 39, 46], it is unknown whether PBSC with HA‐based scaffold and microdrillings would favour hip chondral regeneration for longer than 24–36 months. Therefore, to determine whether intra‐articular injections of PBSC with HA‐based scaffold improved the articular cartilage regeneration in chondral injuries caused by mixed FAIS over a period longer than 24 months post hip arthroscopy.

## MATERIALS AND METHODS

### Study design and setting

For this prospective cohort study, patients were recruited prospectively and nonprobabilistically from a national sports medicine reference centre (MEDS Clinic, Chile). The ethics committee of the Occident Health Service, under No. #02052023 approved this study, which was conducted according to the Helsinki Declaration. All cases satisfied the admissibility criteria and provided written consent to participate.

### Patients

Patients with osteochondral hip injuries classified as grade ≥ IIIB by ICRS [30] or III/IV according to Konan/Haddad classification [23] and mixed‐FAIS who underwent intra‐articular injection of PBSCs with an HA‐based scaffold and micro‐drilling were included between March 2014 and January 2015. The eligibility criteria of patients are summarised in Table 1.

**Table 1:** Eligibility criteria of the study.

Inclusion criteria
1) A mixed FAIS diagnosis defined as a motion‐related clinical disorder of the hip **characterised by** a triad of symptoms, clinical signs, and imaging findings. This **condition** represents symptomatic premature contact between the proximal femur and the acetabulum.
2) ICRS grade ≥ IIIB or III/IV according to Konan/Haddad classification defined as a senior MSK radiologist with more than 20 years of experience.
3) No response to nonsurgical treatment in the last four months.
4) Radiographic and 2.0‐Tesla MRI records; chondral lesion confirmed with standard x‐ray **projections** (posterolateral, axial and crossable) and by 2.0‐Tesla MRI.
5) Surgical indication and general condition compatible with hip arthroscopy.
6) Same surgical team, technique, anaesthetic protocol, and pain management.
7) Patients treated at MEDS clinic (Santiago, Chile).
Exclusion criteria
1) Osteoarthritis (primary radiological signs: joint space narrowing, subchondral sclerosis, subchondral cysts, osteophyte formation, and joint deformities**; s**econdary radiological signs: subluxations and joint effusion).
2) Systemic disease.
3) Chondral lesion size more than 16 mm in diameter.
4) Time between confirmed diagnosis and treatment >6 months.
5) Chondral lesion **caused** by osteochondritis.
6) Hip instability (joint mobility out**side** of physiological passive hip range of motion).
7) Use of intra‐articular corticosteroids in the last six months.
8) Chronic treatment with oral corticosteroids.
9) Concomitant injuries such as dysplasia or previous surgery in the affected hip.
10) Rheumatic disease.
11) Diabetes mellitus.
12) Current or previous hemato‐oncologic disease.
13) Contraindication for filgrastim.
14) Inability to give informed consent.

Abbreviations: FAIS, femoroacetabular impingement syndrome; ICRS, body mass index; MRI, magnetic resonance images; MSK, musculoskeletal.

### Cell therapy

During three consecutive days prior to the surgery, patients received 30 million units (MU)/300 micrograms (μg) of filgrastim in 1 mL (0.3 mg/mL) each day. On the day of surgery, prior to the procedure, a peripheral blood unit (450 mL) was obtained and centrifuged to remove the buffy coat and plasma. The plasma was then centrifuged again at 3500 rpm for 10 min, obtaining a Platelet‐rich plasma (PRP) fraction from the initial blood volume. In parallel, the PBSC‐enriched mononuclear fraction was extracted from the buffy coat by Ficoll density gradient centrifugation at 1800 rpm for 20 min and then re‐suspended in the autologous PRP to a volume ~50 mL (430,000 PBSC ± 270.000 per mL and 640,000,000 ± 110,000 PRP per mL). During the surgical procedure, a fresh dose of ~10 mL from the last obtained volume of ~50 mL with 10% dimethylsulphoxide was applied. The remainder dose of ~40 mL was preserved in cryopreservation (Thermo Fisher Scientific, Inc.) in liquid nitrogen at −195℃ using cryotubes of 5 mL for later use to complete filling of the identified chondral defects as needed.

### Surgery and cell therapy delivery

**Figure 1:**
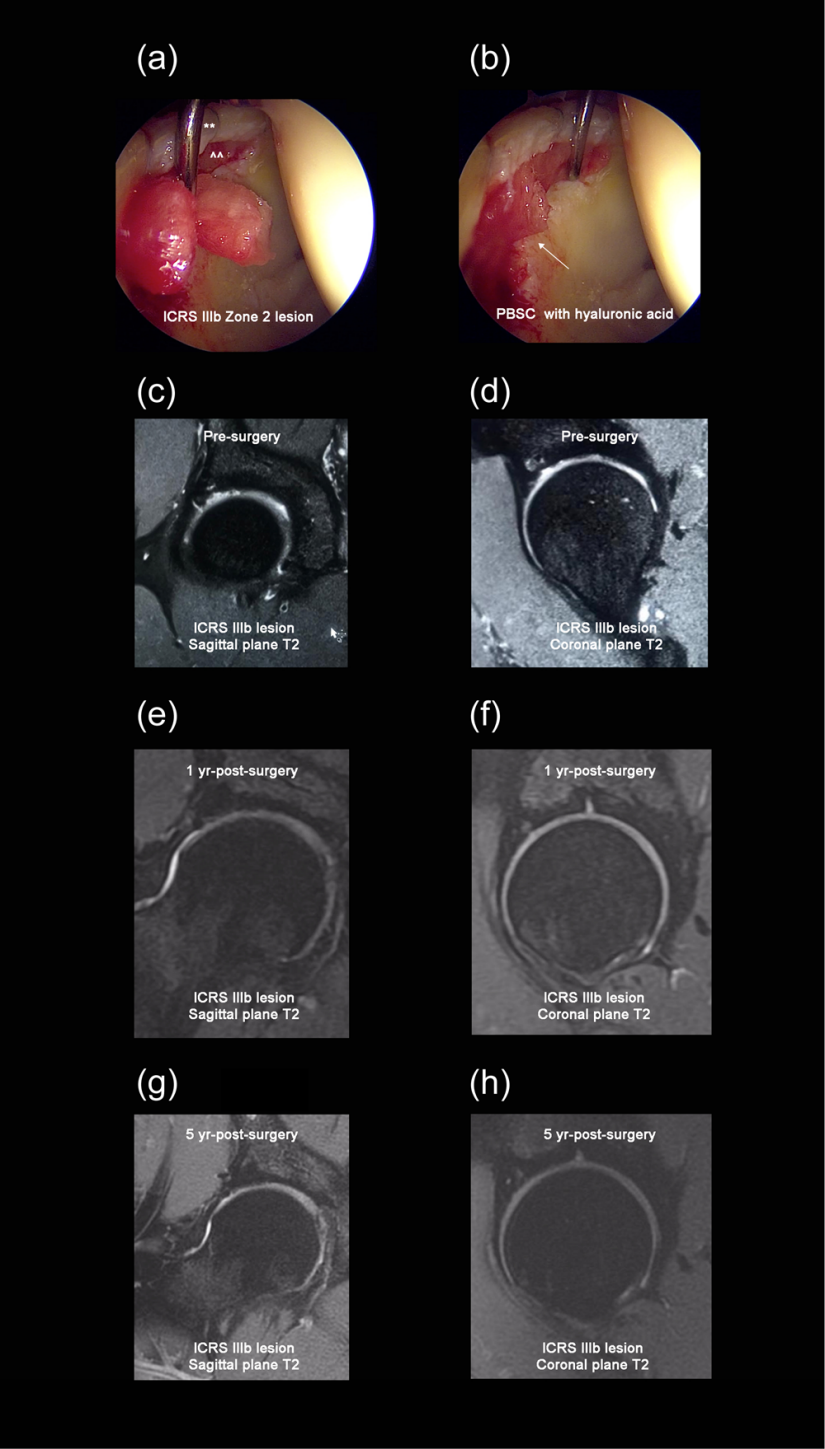
(a) 34‐year‐old male with International Cartilage Repair Society (ICRS) IIIb Zone 2 osteochondral lesion during the placement of the infiltration of peripheral blood stem cells (PBSC) with hyaluronic acid. ** = repaired labrum. ^^ = chondral lesion. (b) Final placement of the PBSC with hyaluronic acid (white arrow) in the chondral lesion for the same 34‐year‐old male patient. (c and d) A T2 sagittal and coronal MRI of a 24‐year‐old male with ICRS IIIb lesion. (e and f) A T2 sagittal and coronal MRI of the same 24‐year‐old male patient at 1 year post‐surgery. (g and h) A T2 sagittal and coronal MRI of the same 24‐year‐old male patient at 5 years postsurgery showed a complete cartilage lesion repaired, stable, and complete subchondral bone repaired.

Hip arthroscopy was developed under spinal anaesthesia, without traction, and an out‐in approach [40, 41]. For diagnostic purposes, only a longitudinal capsulotomy was performed to access the joint. Once the joint was accessed, we used three classic portals to operate on the FAIS [10] to repair the labrum tear, pincer and/or CAM deformity. After the repair, the chondral lesion was measured and delimited by debriding (Smith & Nephew PLC) the damaged cartilage until healthy cartilage tissue was reached. The calcified plaque was resected using a curette (Smith & Nephew PLC). Thereupon, micro‐drilling was performed, assisted through a curve guide at low speed, with 1.2 mm bit, reaching a depth of 7 mm, and forming a stable bone bridge. The next step was to infuse 20 mL of air to visualise a dry chondral lesion. At this point, 10 mL of PBSC was suspended in the HA‐based scaffold (hyalofast®, Anika Therapeutics, Inc.), and the PBSC was fitted into the chondral lesion (Figure 1). We sutured the capsule to end the surgery, performing three side‐to‐side repairs using nonabsorbable sutures (Smith & Nephew PLC). Finally, 5 mm of intra‐articular PBSC was delivered. All procedures were performed by the same senior surgeon CM, and assistants CR and CS. The surgeons had more than 15 years of experience in hip surgery.

### Postsurgery protocol

In the acute phase, nonsteroidal anti‐inflammatory drugs (300 mg of ketoprofen in 500 mL of Ringer's solution) were administered at 10 mL/h for 24 h. In addition, the patients were prescribed 1 g of acetaminophen every eight hours for 15 days. The patients received Rivaroxaban 10 mg daily and naproxen 550 mg daily for 21 days for thromboembolism and heterotopic calcification prevention. No epidural blocks, femoral blocks, or possible opioid rescue were used.

All patients underwent the same rehabilitation protocol. The first stage aimed for hip protection and range‐of‐motion exercises with partial weight bearing for four weeks. The intermediate stage aimed to increase the hip range‐of‐motion, flexibility, and hip stabiliser muscle strength over the next eight weeks. The patients were instructed to avoid running, jumping, and any physical activity involving landing tasks for up to one‐year postsurgery to prevent increased impact forces [4]. The last stage aimed at sports‐specific training without running or jumping until one‐year postsurgery. Patients were allowed to return to full sports participation after one‐year postsurgery.

### Outcomes: Imaging and clinical assessment

Magnetic resonance imaging (MRI) was performed with a 2.0‐Tesla device. Radiologists were blinded to the patient data and were unaware of the purpose of the study. The radiology assessment included criteria such as the repaired cartilage signal, morphological characteristics of the repaired tissue, the integration of the tissue into the borders, and the presence of subchondral oedema [51]. All MRI procedures used the T2 Mapping software [3]. The newly formed tissue repair was quantified using the ICRS morphologic score system (MSS) [1, 23, 30, 42, 44]. The MSS, which is the primary outcome of the study, was measured prior to the surgery and in the fifth year. We decided to use the MSS developed by Mithoefer et al. [31] and developed by the ICRS [30] because it allows effective and reproducible quantification, especially of the amount and quality of the neoformed chondral tissue.

The clinical evaluation was obtained using both the Hip Outcome Score (HOS) [27] and the Visual Analogue Scale for Pain (VAS Pain) [18]. The HOS evaluates hip functionality on a scale from 0% to 100%, where 100% is the higher score. The HOS was measured prior to the surgery, at month six, and one and five years after the surgery. The VAS Pain evaluates the level of pain perception using a scale of 100 mm, where 0–4 mm indicates no pain, 5–44 mm indicates mild pain, 45–74 mm indicates moderate pain, and 75–100 mm indicates severe pain. The VAS Pain was measured prior to the surgery and in the fifth year.

All clinical assessments were previously validated in the native language of patients [13, 43], and the radiological chondral repair scale was previously validated [44].

### Statistical analysis

We described the largest diameter of injury, the median follow‐up, the proportion of clinical and radiological scores improvements, and complications. The MSS, HOS, and VAS Pain were tested for normality using the Shapiro–Wilk test. In consequence, the data are presented as mean and standard deviation. *T*‐test, within‐subjects ANOVA, and multiple comparisons were applied to analyse the time effect. The intra‐class coefficient correlation (ICC) for MSS, which was repeated once by the same evaluator, was estimated. The type error I was set to 5%. Statistical analyses were performed using SPSS 2019 (IBM Crop.).

The minimal clinically important difference that represents the smallest improvement considered worthwhile by a patient [7] was a change of 20 mm for the VAS Pain scale. Regarding the HOS, we have considered a score of 7.9 of change based on hip arthroscopy revision data [33]. Finally, we considered a 3‐point improvement for the MSS based on histological images showing cartilage healing improvement in the femoral trochlea with osteochondral defects in rabbits [19].

The *posteriori* power‐size estimation and effect size for the minimal significant (*p* < 0.05) multiple comparisons was described. The power estimations were made using G*Power software (Düsseldorf, Mannheim, and Kiel University).

## RESULTS

The median follow‐up was 5.1 ± 0.3 years. Of the initially 34 patients, seven were excluded due to incomplete MRI records, and there were two dropouts. Thus, 25 patients (5 women and 20 men; mean age of 34.9 ± 9.2 years at the time of surgery, body mass of 72.3 kg, body mass index of 26.4 ± 9.2 kg m^−2^, and physical activity at least five times per week) were finally studied (Table 2). The locations and size characteristics of the chondral lesions are summarised in Table 3. The typical arthroscopy and MRI characteristics observed in patients during the follow‐up are summarised in Figure 1.

**Table 2:** Basal characteristics of the patient (*n* = 25).

Age, mean ± SD, years	34.9 ± 9.2
Body mass, mean ± SD, kg	78.3 ± 8.3
BMI, mean ± SD, kg/m^2^	26.4 ± 3.1

Abbreviations: BMI, body mass index; SD, standard deviation.

**Table 3:** Hip chondral lesions characteristics (*n* = 25).

	Number of patients (No.)	MSS classification (Pts)	Konan & Haddad classification (Pts)	Diameter (mm)
**Zone 2**	8	3.5 ± 0.2	3.2 ± 0.3	12.4 ± 3.1
**Zone 3**	14	3.3 ± 0.3	3.6 ± 0.2	13.5 ± 2.8
**Zone 4**	3	3.7 ± 0.1	3.6 ± 0.3	11.4 ± 1.9

Abbreviation: MSS, The International Cartilage Repair Society morphologic score system.

Only one patient (1/25, 4%) reported a few side effects: myalgia and fever during the filgrastim administration. No infection, tumours, or synovitis was reported at the end of the follow‐up.

**Figure 2:**
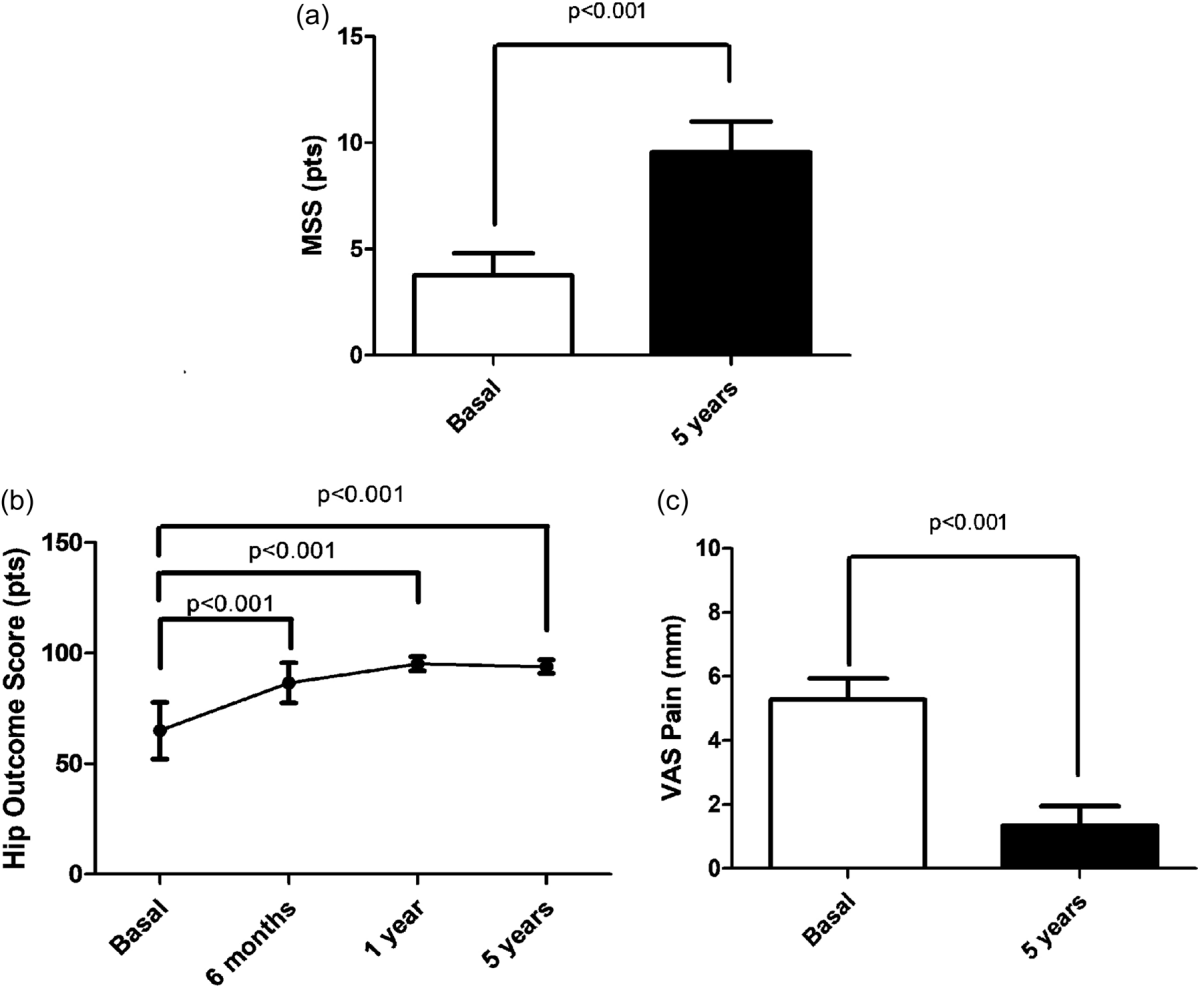
(a) The International Cartilage Repair Society morphologic score system (MSS) at basal (presurgery) and 5 years postsurgery. (b) Change in Hip Orthopaedic Score (HOS) at basal (presurgery), at 6 months, 1 and 5 years postsurgery. (c) The Visual Analogue Scale for Pain (VAS Pain) at basal (presurgery).

Ninety‐two percent (23/25) of the patients improved their outcomes. The MSS increased from 3.8 ± 1.1 pts to 9.6 ± 1.5 pts (*p* < 0.001) (Figure 2). The HOS increased from 65.5 ± 13.0 pts, to 86.2 ± 9.1 pts, to 95.0 ± 2.8 pts, to 93.9 ± 2.4 pts (*p* < 0.001), respectively (Figure 2). The VAS‐Pain decreased from 5.3 ± 0.7 to 1.3 ± 0.6 mm (*p* < 0.001), Figure 2. The improvements for MSS, HOS, and VAS Pain were greater than >3 pts, >7.9 pts, >20 mm (minimal clinically important difference), respectively. The obtained power‐size was 0.99 with an effect size of 0.92 for the multiple comparisons obtained for the HOS variable (basal vs. six months). The obtained ICC for MSS at basal and fifth‐year values was 0.93 (95% CI: 0.85–0.93) and 0.95 (95% CI: 0.89–0.98), respectively.

## DISCUSSION

Our most important finding in patients with mixed‐FAIS over 5 years was that the intra‐articular injection of PBSC suspended in HA‐based scaffold and micro‐drillings improves (1) the cartilage regeneration of the hip chondral lesions, (2) the no deterioration of the neoformed tissue, (3) pain perception, and (4) functionality and independence in daily activities after chondral lesions. Furthermore, 92% (23/25) of the patients returned to sports practice. Additionally, our results were clinically important and consistent with PBSC and HA infiltration after microfracture in damaged knees [42]. As far as we explored, this is the most extended prospective follow‐up using PBSC suspended in an HA‐based scaffold.

Our study suggests that PBSCs with HA‐based scaffold and micro‐drilling techniques could confer biological stability at the chondral lesion and favour the differentiation of stem cells [42, 48], resulting in stable neoformed cartilage tissue after 5 years. This neoformed cartilage tissue was consistent with previously reported knee chondral regeneration [42] and comparable with the efficacy of bone marrow cell use [11, 38, 39, 46]. Although the exact underlying mechanisms and modulation of cartilage repair are unknown, there appears to be increased expression of growth factors such as platelet‐derived growth factor [28, 32, 50]. For example, traumatic injury forms a platelet‐rich haematoma, which releases growth factors and initiates inflammatory cell recruitment. These inflammatory cells release additional growth factors and cytokines that continue healing process. In this regard, PBSCs may have facilitated tissue healing [49] through the self‐renewal, multipotentiality, and plasticity of stem cells [6, 22]. Furthermore, in vitro studies have shown that PBSCs with HA exhibit the capability for restorative healing, inflammation modulation, and cartilage repair, increasing cell proliferation via cartilage‐derived morphogenetic protein 2 in conjunction with insulin‐like growth factor (IGF‐1), transforming growth factor beta‐1 (TGF‐b1), and platelet‐derived growth factor [2]. Hence, the PBSCs with an HA‐based scaffold technique could fill the lesion by capturing stem cells, supporting their attachment, proliferation, and differentiation [45]. The release of HA into the lesion may have created an embryonic‐like microenvironment to promote cartilage growth [45].

This study observed no deterioration of the neoformed cartilage following 5 years of PBSCs suspended in the HA‐based scaffold and micro‐drillings. This finding suggests that the neoformed cartilage tissue is of better quality than that produced by microfractures alone, which is the recommended technique for regenerating the chondral lesions [4, 29, 31]. Unfortunately, microfractures alone are associated with neoformed tissue deterioration, which can be present until 24 months post‐surgery [8, 9, 31]. Moreover, microfracture alone can also result in subchondral bone deterioration or fracture [30, 31]. In contrast, our patients achieved stable neoformed tissue after 5 years postsurgery (Figure 1). We infer that our developed micro‐drilling technique has an advantage over classical microfractures because micro‐drilling may have reduced the subchondral damage [5]. This advantage allows for better control of the excessive cell invasion from the bone marrow bleeding in Fortier channels when deep drills (~8 mm) are performed [24]. Therefore, preserving stable subchondral bone is another essential goal for achieving better neoformed tissue. Otherwise, a fibrocartilaginous tissue fills the chondral defect, which tends to deteriorate faster [32, 42, 48].

The lower perception of pain and improved functional hip scores are clinically important findings because they suggest enhanced joint function. This suggests the clinical relevance of using PBSCs suspended in HA‐based scaffold with micro‐drilling as a therapeutic option for chondral hip lesions. Our outcomes align with those reported after hip preservation surgeries in a meta‐analysis involving 1502 operated hips, with a mean age of 38.0 ± 1.3 years and mean follow‐up of 31.8 ± 9.6 months [34].

Finally, stem cells offer many advantages [6, 22] and can easily be acquired, expanded, and used for cartilage injuries. Stem cells have been extensively studied in vitro and in vivo research [1, 11, 16, 21, 35, 48]. In this study, we chose to use PBSCs obtained from peripheral blood because the process (apheresis) is straightforward, cost‐effective, mostly automated, and associated with a reduced risk of contamination. The technique requires the administration of filgrastim, a drug with very low toxicity that is extensively used in bone marrow transplants and approved by the United States Food and Drug Administration. Filgrastim is a recombinant protein that stimulates the growth of white blood cells. In our series, only one patient reported a few side effects, such as myalgia and fever, during the filgrastim administration. Another potential disadvantage of using stem cells is the risk of hypertrophy or tumorigenesis [22]. However, in our study, we did not observe cartilage hypertrophy on radiological images, nor were there any benign or malignant tumours, either locally or systemically.

Our study is not out of limitations. The main limitation was the absence of a control group, which is necessary for a clinical trial with a randomised method to confirm our findings. In addition, a higher period of follow‐up period might be most appropriate that is, 10 years of follow‐up and MRI re‐evaluation may have been preferable. Moreover, our functional outcomes could be further complemented by biomechanical testing to assess hip recovery tissue properties, but unfortunately, we were unable to conduct the assessment for all patients. MRI magnets with less than 3T strength could benefit from arthrogram injection. Unfortunately, our study lacked biopsies due to insufficient funding. Although the sample size is relatively small, the study possesses high statistical power and is likely the longest‐reported study with novel outcomes and techniques for hip preservation literature.

## CONCLUSION

The intra‐articular administration of PBSCs with HA‐based scaffold and micro‐drilling promotes chondral regeneration and provides evidence of clinical stability without deterioration of the neoformed tissue. It also decreased the perception of pain, and increased hip functionality and independence in daily activities following chondral lesions associated with a mixed‐FAIS over 5 years of evolution. However, a clinical trial is required to confirm our promissory findings.

## AUTHOR CONTRIBUTIONS

*Conceptualisation*: Juan Eduardo Monckeberg and Claudio Rafols. *Methodology*: Juan Eduardo Monckeberg and Claudio Rafols. *Software*: Leticia Del Canto. *Validation*: Juan Eduardo Monckeberg, Claudio Rafols, Philipp Gerhard, Leticia Del Canto, Julio Rosales, Marco Antonio Verdugo, Cristobal Saez, and Carlos De la Fuente. *Formal analysis*: Carlos De la Fuente and Juan Eduardo Monckeberg. *Investigation*: Juan Eduardo Monckeberg, Claudio Rafols, Philipp Gerhard, Leticia Del Canto, Julio Rosales, Marco Antonio Verdugo, and Cristobal Saez. *Resources*: Juan Eduardo Monckeberg and Leticia Del Canto. *Data curation*: Juan Eduardo Monckeberg and Leticia Del Canto. *Writing–original draft*: Juan Eduardo Monckeberg and Carlos De la Fuente. *Writing–review and editing*: Carlos De la Fuente. *Visualisation*: Carlos De la Fuente. *Supervision*: Juan Eduardo Monckeberg, Claudio Rafols, Philipp Gerhard, Leticia Del Canto, Julio Rosales, Marco Antonio Verdugo, and Cristobal Saez. *Project administration*: Juan Eduardo Monckeberg. *Funding acquisition*: None.

## CONFLICT OF INTEREST STATEMENT

The authors declare no conflict of interest.

## ETHICS STATEMENT

The ethics committee of the Occident Health Service (Santiago, Chile), under No. #02052023 approved this study, which was conducted according to the Helsinki Declaration. All cases satisfied the admissibility criteria and provided written consent to participate.

## Data Availability

Data will be available directly from Dr. C. Monckeberg.
